# Prevalence of hepatitis C infection among the general population and high-risk groups in the EU/EEA: a systematic review update

**DOI:** 10.1186/s12879-019-4284-9

**Published:** 2019-07-23

**Authors:** Ru Han, Junwen Zhou, Clément François, Mondher Toumi

**Affiliations:** 1 University of Aix-Marseille, Marseille, France; 2 Creativ-Ceutical, 215, rue de Faubourg St-Honoré, 75008 Paris, France

**Keywords:** Hepatitis C, Prevalence, Europe, People who inject drugs, Men who have sex with men, Prisoners, High-risk groups, Systematic review

## Abstract

**Background:**

Although significant improvement in efficacy measured by a sustained virological response, the high acquisition costs of direct-acting antivirals limit the access for patients and influence the costs of healthcare resource utilisation in hepatitis C. It is important to have the latest estimates of prevalence, especially in high-risk groups, for cost of illness, cost-effectiveness and budget impact studies.

**Methods:**

Original studies on the estimates of the prevalence among general and high-risk groups in the European Union/European Economic Area (EU/EEA) were retrieved from Medline and Embase for the period from 2015 to 2018. All included studies were evaluated for risk of selection bias and summarised together in a narrative form. Results from previous reviews and updated searches were compared per country among different populations, respectively.

**Results:**

Among the 3871 studies identified, 46 studies were included: 20 studies were used for the estimate of the general population; 3 for men who have sex with men (MSM); 6 for prisoners; and 17 for people who inject drugs (PWID). Compared with the results reported in previous systematic reviews, the updated estimates were lower than previously in most available countries. Anti-HCV general population prevalence estimates ranged from 0.54 to 1.50% by country. The highest prevalence of anti-HCV was found among PWID (range of 7.90–82.00%), followed by prisoners (7.00–41.00%), HIV-positive MSM (1.80–7.10%), HIV-negative MSM (0.20–1.80%), pregnant women (0.10–1.32%) and first-time blood donors (0.03–0.09%).

**Conclusions:**

Our study highlights the heterogeneity in anti-HCV prevalence across different population groups in EU/EEA. The prevalence also varies widely between European countries. There are many countries that are not represented in our results, highlighting the need for the development of robust epidemiological studies.

**Electronic supplementary material:**

The online version of this article (10.1186/s12879-019-4284-9) contains supplementary material, which is available to authorized users.

## Background

Infection with hepatitis C virus (HCV) leads to an asymptomatic acute stage. However, approximately 75% of acutely infected patients face a substantial risk of developing chronic HCV infection [1]. During the 2 decades after infection, 27% develop liver cirrhosis, and 25% develop hepatocellular carcinoma (HCC) [2, 3]. Worldwide, an estimated 71 million people were living with chronic HCV infection (1.0% of the global population) [4]. Whilst, in the European Union/European Economic Area (EU/EEA), it was estimated that more than 14 million people were living with chronic HCV infection, suggesting a relatively higher prevalence of 1.5% in this region [4].

Given to the slow rates of liver disease progression, many countries are yet to experience the full burden of HCV-related disease [5]. However, decade-long delays between infection and the expression of chronic liver disease or liver cancer made it difficult to link these diseases to earlier HCV infections. Reliable and timely prevalence data is therefore important to describe the current burden of the disease.

Most people infected with HCV remain unaware of their infection. The hidden burden estimated, based on limited data from the EU/EEA, shows that less than 15% of those chronically infected with HCV are aware of their diagnosis [6–8]. An anti-HCV antibodies serology test is recommended by the European Association for the Study of the Liver (EASL), as the first-line diagnostic test for HCV screening, which is evident of the past or current HCV infection [9]. If the result is positive, then the current infection should be confirmed by a sensitive RNA test. Anti-HCV antibodies are detectable by enzyme immunoassay (EIA) in the vast majority of patients with an HCV infection. In addition, rapid diagnostic tests (RDTs) are also recommended in settings where there is limited access to laboratory infrastructure and testing or populations where access to RDTs would facilitate linkage to care [10]. The primary goal of diagnostic testing is to identify and link infected individuals to appropriate treatment. Several modelling studies suggest that scaling up an HCV treatment can lead to substantial reductions in anti-HCV prevalence and reduce transmission [11–14]. The introduction of direct-acting antivirals (DAAs) has been a major breakthrough in the treatment of hepatitis C. However, the high acquisition costs of sofosbuvir-based regiments limit the access for patients and influence the costs of healthcare resource utilisation in hepatitis C [15]. It is important to have the latest estimates of prevalence, especially in high-risk groups, for cost of illness, cost-effectiveness and budget impact studies.

This study chose the most published reviews with a low risk of selection bias, according to an overview of systematic reviews on clinical burden of HCV infection [16]. This study updated 2 previous systematic reviews undertaken respectively among the general population [17] and high-risk groups [18] in 2015. In Europe, the high-risk groups for the acquisition of HCV include people who inject drugs (PWID), men who have sex with men (MSM) and people in prison. The aim of this study was to update and expand the estimates for anti-HCV prevalence.

## Objectives

The objective of the study is to update the anti-HCV prevalence (the serologic markers used as proxies for chronic infection in this study) among the general population and high-risk populations (MSM, prisoners, and PWID).

## Methods

### Date source and search strategy

Original research studies on the estimates of the prevalence among general and high-risk populations in the EU/EEA were retrieved from Medline and Embase for the period from 2015 to 2018. The search strategy used was consistent with previous reviews [17, 18] and is shown in Additional file 1. The search terms covered the following domains: disease-related (HCV infection), outcome-related (anti-HCV/HCV RNA prevalence), and geographic-related search terms (EU/EEA). Two separate searches were conducted to maximise the yield of the search, so that no population-specific search terms were included among the general population, MSM and prisoners. However, PWID-specific terms were included due to 2 reasons. The first being that previous reviews didn’t conduct literature database searching, whilst the second was because the result of prevalence among PWID was only from the European Monitoring Centre for Drugs and Drug Addiction (EMCDDA). The relevant yield among PWID was much according to our preliminary search. To cover the complete time scope of the published studies the search for the general population, MSM and prisoners was limited to records between January 2015 and December 2018 and the search for PWID was limited between January 2009 and December 2018.

### Inclusion/exclusion criteria and data extraction

The inclusion/exclusion criteria [17, 18] considered population, outcomes of interest, study designs, publication timeframe, and geographical scope. Studies were included if they: 1) reported anti-HCV seroprevalence among the general population, pregnant women, first-time blood donors, PWID, MSM, or prisoners; 2) measured the actual presence of viral markers (anti-HCV antibody in this study) in bodily fluid or dried blood spot samples in human subjects; 3) reported original data; 4) were published after 2015 to the present among the general population, pregnant women, first-time blood donors, MSM, or prisoners and published after 2009 to the present among PWID; 5) reported outcomes in one or more EU/EEA member states or any of their regions. Studies were excluded if they 1) targeted non-representative populations, e.g. the homeless, migrants, patients with specific diseases etc.; 2) did not report specified serological markers, or the reported study was not conducted on humans or only a self-reported/unconfirmed prevalence; 3) reported modelled or extrapolated data only, or opinion papers, editorials, guidelines or recommendations, correspondence articles, systematic reviews or meta-analysis; 4) were published out the targeted timeframe; 5) reported data on non-EU/EEA countries only. More details on the inclusion/exclusion criteria are shown in Additional file 1.

The extraction form included year, country, characteristics of the analysed population, the sampling method, laboratory test used, participation rate, number of participants, and anti-HCV results. For studies reporting the prevalence in MSM, data on HIV sero-status was also extracted.

### Quality assessment for risk of selection bias

Each included study was evaluated for risk of selection bias using frameworks developed by Hofstraat et al. and Falla et al. [17, 18]. For studies among the general population and prisoners, three domains were included: whether estimates were standardised by age and sex, the representativeness of sampling (e.g. random vs. convenience sampling) and geographical coverage. For PWID and MSM studies, just one domain was included: geographical coverage. Points were awarded in each domain for a lower risk of bias, and a total score calculated by summing the values in each domain. An estimate among the general population and prisoners was considered of low risk when it reached a study quality score ≥ 4. A low risk estimate of prevalence in PWID and MSM was defined as a study quality score ≥ 1.

### Data analysis

All included studies were summarised together in a narrative form and in summary tables that tabulate the important description of the study population, recruiting period, results and study quality. According to the results of the systematic review previously performed, an algorithm was applied to different populations separately [17, 18]: If a single low risk of selection bias prevalence estimate was available for a country, this was used. If a low risk of selection bias estimate was not available, high risk of selection bias estimates were used (these were pooled when possible). Data per country were pooled according to the standard error and sample size. Then 95% confidence intervals were calculated by the estimated average and pooled sample size. Results of subgroup analyses of age-specific, gender-specific, or injecting risk-specific prevalence were reported. Results from previous reviews and the updated searches were compared per country among different populations, respectively.

The overall population was categorised into 2 groups: (1) the general population, inclusive of susceptible population with no recognised risk factors for reinfection (communities, screening participants, pregnant women, and first-time blood donors); (2) high-risk populations, inclusive of susceptible MSM, prisoners, and PWID. The majority of EU/EEA countries offer antenatal HCV screening and first-time blood donors screening. These 2 subgroups among the general population are the most complete population prevalence source and used as a proxy population [17]. However, we conducted separate analysis between pregnant women, first-time blood donors and other general populations because the previous systematic review reported that they are not representative enough.

Some estimates among the general population included exclusively multiple subgroups, overlapping with high-risk populations. When pooled together, however, the subgroup data were excluded and pooled with the relevant high-risk populations based on the results of quality assessment.

## Results

### Literature search retrievals

**Fig. 1:**
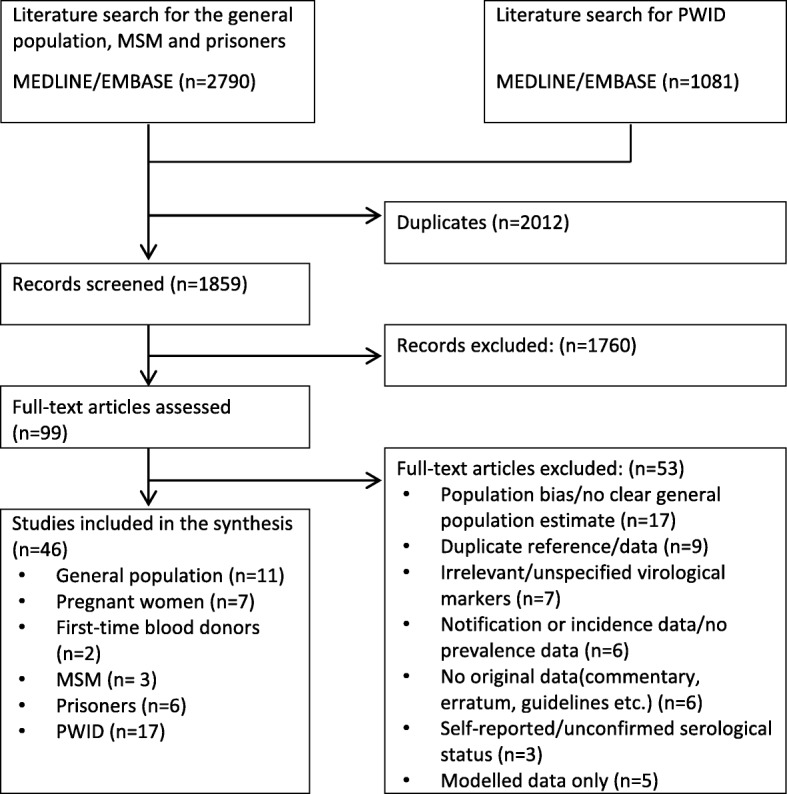
Flow diagram of the study selection process

The search for data on anti-HCV prevalence in the general population and MSM, and people in prison and PWID identified 2790 and 1081 citations, respectively. After the title/abstract screening, 73 articles for the general population with 2 subgroups and 26 articles for PWID were included. Following the full text screening of these 99 papers, 53 publications were considered not relevant. Finally, 46 publications were included in the review of prevalence data, with 11 publications used for the estimate of the general population, 7 for pregnant women, 2 for first-time blood donors, 3 for MSM, 6 for prisoners and 17 for PWID (Fig. 1). The results of quality assessment for risk of selection bias are shown in Additional file 1.

### Anti-HCV prevalence among the general population in EU/EEA

#### General population

The anti-HCV general population prevalence estimates were found for 7 of the 34 countries in our review, ranging from 0.54 to 1.50% by country (Table 1). More than one estimate was available for 4 countries of the 7 countries covered, with the most estimates for Spain (*n* = 3). Eight low risk of selection bias estimates from 5 countries (Czech Republic, Ireland, Italy, Portugal and Spain) were available. Multiple low-risk of selection bias estimates were available for a pooled estimate in Italy (*n* = 2). A relatively high anti-HCV prevalence was found in the Czech Republic (1.67%), Poland (1.50%) and Italy (1.37%). The estimate for Poland, however, is based on one single study with a high risk of selection bias (score = 2). The other article reporting the prevalence estimate on the general female population in Poland was not pooled. Three estimates were available for Spain, of which only one was of low risk of selection bias and reported an anti-HCV prevalence of 1.11%. One article reported 1.14% in the Callosa D’En Sarrià and Valencian Region. The other one reported 0.60% in the general female population. Subgroup analysis of age-specific prevalence was available for Italy [22, 23] and Poland [24]. In Italy [22, 23], the prevalence of HCV increased with an increasing birth cohort (0.20% in subjects born after the year 1984, 1.20% in those born in 1975–1984, 1.60% in those born in 1965–1974, 1.20% in those born in 1955–1964, 2.20% in those born in 1945–1954, 7.00% in those born in 1935–1944, and 4.20% in those born before the year 1953). In Poland [24], a higher prevalence was found in the post-reproductive population with an age more than 45 years (1.50% in 15–24 years, 1.20% in 25–34 years, 1.60% in 35–44 years, 2.90% in 45–54 years and 2.60% in 55–64 years).

**Table 1:** Summary of results from studies reporting anti-HCV prevalence in the general population

Author, year	Country	Recruiting period	Population as reported	Age, mean (SD)	Sample size	Anti-HCV prevalence (%) (95% CI)	Risk of selection bias
Viejo, 2018 [19]	Spain	February–April 2017	The general adult population living in the health area of Callosa D’En Sarrià	47.5 (−)	2637	1.14 (0.73–1.55)	High risk
Lavin, 2017 [20]	Spain	2015–2016	Spanish adult population	–	6839	1.11 (−)	Low risk
Quesada, 2015 [21]	Spain	1994–2005	Women from the general population in different geographical areas worldwide	40.0(15.6)	314	0.60 (0.20–2.50)	High risk
Andriulli, 2018 [22]	Italy	2015	The general population	–	4907	2.30 (−)	Low risk
Morisco, 2017 [23]	Italy	May 14	A random 1:3 systematic sample of the adult general population of Naples	49.9(5.00)	1315	3.00 (2.10–4.00)	Low risk
Walewska-Zielecka, 2017 [24]	Poland	2004–2014	Patients who had been tested for anti-HCV at least once in the period from 2004 to 2014	34.4(8.6)	61805	1.50 (−)	High risk
Clifford, 2017 [25]	Poland	2004–2009	The general female population	37.0(−)	909	0.80 (0.30–1.60)	High risk
Garvey, 2017 [26]	Ireland	April–June 2014 and November 2015–February 2016	The adult population in Ireland with probability proportional to the general population age-sex distribution	–	3759	0.98 (−)	Low risk
Chlibek, 2017 [27]	Czech Republic	February 2015–September 2015	The adult general population	47.1(17.1)	3000	1.67 (1.27–2.19)	High risk
Carvalhana, 2016 [28]	Portugal	April 2012–December 2014	Adults from primary care settings in mainland Portugal	50.2(18.3)	1627	0.54 (0.20–0.90)	Low risk
Plompen, 2015 [29]	Netherland	−	The general Dutch elderly population	69.5(9.0)	6036	0.56 (−)	High risk

#### Pregnant women

An estimate of antenatal anti-HCV prevalence was found for 6 of the 34 countries in our review, ranging from 0.10 to 1.32% by country (Table 2). More than one estimate was available only for the UK of the 6 countries covered (*n* = 2). Five low risk of selection bias estimates from 5 countries (Poland, Slovenia, Spain, Sweden and the UK) were available. Only the estimate of Italy was of high risk of selection bias. Relatively high anti-HCV prevalence was found in Poland (1.32%) and Spain (0.55%). Relatively low prevalence was reported in the UK (0.10%). The other article reporting the prevalence estimate on women who attended antenatal clinics in London (0.5%) was not pooled given to the potential geographic bias. Subgroup analysis of age-specific prevalence was available for the UK [30, 31], Poland [34] and Slovenia [32]. In the UK [30, 31], the antenatal anti-HCV prevalence increased with increasing age in mothers born in the UK (0.00% in younger than 21 years, 0.00% in 21–25 years, 0.00% in 26–30 years, 0.03% in 31–35 years and 0.07% in older than 35 years) and Asia-Pacific (0.00% in younger than 21 years, 0.00% in 21–25 years, 0.12% in 26–30 years, 0.16% in 31–35 years and 0.49% in older than 35 years), but peaked in mothers of 26–30 years (0.85%) born in Eastern Europe (0.00% in younger than 21 years, 0.22% in 21–25 years, 0.16% in 31–35 years and 0.29% in older than 35 years). No statistically significant differences in antenatal anti-HCV prevalence between age groups were reported in Poland (0.90% in 15–24 years, 0.70% in 25–34 years and 0.80% in 35–44 years) [34] and Slovenia (0.00% in younger than 20 years, 0.07% in 20–29 years and 0.05% in older than 30 years) [32].

**Table 2:** Summary of results from studies reporting anti-HCV prevalence in pregnant women and first-time blood donors

Author, year	Country	Recruiting period	Population as reported	Age, mean (SD)	Sample size	Anti-HCV prevalence (%) (95% CI)	Risk of selection bias
Pregnant women							
Orkin, 2016 [30]	UK	2013	Women who attended antenatal clinics during 2013 at 2 London hospitals	–	1000	0.50 (0.06–0.94)	High risk
Cortina-Borja, 2016 [31]	UK	1 April–30 June 2012	Women delivering live-born infants in the North Thames region in England	–	31467	0.10 (0.07–0.14)	Low risk
Kopilovic, 2015 [32]	Slovenia	1999, 2003, 2009 and 2013	Pregnant women	–	31849	0.13 (0.09–0.17)	Low risk
Lembo, 2017 [33]	Italy	January 2010–December 2015	Pregnant women consecutively admitted to the Division of Obstetrics and Gynaecology of the University Hospital of Messina, Italy	–	5184	0.20 (−)	High risk
Walewska-Zielecka, 2016 [34]	Poland	2004–2014	Pregnant women in Poland	33.4(7.9)	42274	1.32 (−)	Low risk
Millbourn, 2017 [35]	Sweden	October 2013–March 2015 and October 2013–February 2016	Every pregnant woman and her partner in Orebro county and in Southern part of Stockholm (288,000 and 300,000 inhabitants, respectively)	–	21379	0.20* (−)	Low risk
Munoz-Gamez, 2016 [36]	Spain	January–October 2015	Pregnant women in Spain	–	−	0.55 (0.55–0.77)	Low risk
First-time blood donors							
Velati, 2018 [37]	Italy	January 2009–December 2015	Voluntary, unpaid first-time donors	–	1934612	0.09 (0.08–0.09)	Low risk
Politis, 2018 [38]	Greece	2010–2016	Blood donor	–	−	0.03 (−)	High risk

*calculated based on available data

#### First-time blood donors

An estimate of anti-HCV prevalence in first-time blood donors was available for only 2 of the 34 countries in our review, with Greece reporting 0.03% and Italy reporting 0.09% (Table 2). One high risk of selection bias estimate for Greece and one low risk of selection bias estimate for Italy were available. There is no subgroup analysis among first-time blood donors reported.

### Anti-HCV prevalence among high-risk populations in the EU/EEA

#### MSM

An estimate of anti-HCV prevalence in MSM was found for 3 of the 34 countries in our review (Table 3). Furthermore, the MSM was divided into 2 categories: HIV-positive MSM and HIV-negative MSM. The prevalence in HIV-positive MSM covered 3 countries, ranging from 1.80% (the UK) to 7.10% (the Netherlands). The other country, France, reported 5.10%. The prevalence in HIV-negative MSM covered 2 countries, ranging from 0.20% (the UK) to 1.80% (France). Two low risk of selection bias estimates from 2 countries (the UK and France) were available. Only the estimate of the Netherlands was of high risk of selection bias. There is no subgroup analysis among MSM reported.

**Table 3:** Summary of results from studies reporting anti-HCV prevalence in MSM and prisoner

Author, year	Country	Recruiting period	Population as reported	Age, mean (SD)	Sample size	Anti-HCV prevalence (%) (95% CI)	Risk of selection bias
MSM							
Ireland, 2017 [39]	UK	28 February - 15 December 2014	MSM attending 4 genitourinary medicine clinics in Manchester	–	HIV+:735 HIV-:855	1.80 (−) 0.20 (−)	Low risk
Vanhommerig, 2013 [40]	Netherland	2009–2012	HIV-infected MSM during 5 waves of anonymous surveys at Amsterdam STI clinic	–	439	7.10 (−)	High risk
Cotte, 2018 [41]	France	January 2016 to May 2017	HIV+, HCV-negative MSM with serological follow-up in 2016	–	HIV+:13051 HIV-:930	5.10 (−) 1.80 (−)	Low risk
Prisoner							
Ekeke, 2018 [42]	UK	December 2015-February 2017	Prisoners entered Pentonville prison	–	1324	7.00 (−)	High risk
Patel, 2016 [43]	UK	−	Inmates in a medium security prison	–	160	33.75 (−)	High risk
Casella, 2016 [44]	Portugal	2014 and 2016	Inmates of 2 male prisons in the centre of Portugal (Pinheiro da Cruz and Setubal)	–	82	38.00 (−)	High risk
Liberal, 2017 [45]	Portugal	January–April	Inmates from one of the largest prisons in Portugal	–	1208	15.70* (−)	High risk
Svendsen, 2017 [46]	Norway	September 2015	At-risk populations in Trondheim, Norway	–	304	41.00 (−)	High risk
Lerena, 2016 [47]	Spain	−	Inmates in a Northern region of Spain (Cantabria) with 600 k inhabitants and focused on the regional long-stay prison of El Dueso	–	436	16.00 (−)	High risk

#### Prisoners

An estimate of anti-HCV prevalence was found for 4 of the 34 countries in our review, ranging from 7.00 to 41.00% by country (Table 3). More than one estimate was available for the UK (*n* = 2) and Portugal (*n* = 2) of the 4 countries covered. All estimates in prisoners were of high risk of selection bias. All studies were single-centre in a regional level, except the one in Portugal, which was a multi-centre. Three studies reported a ratio of sex with more males than females. The other 3 studies did not report a ratio of sex. None of these 6 studies reported data on age. The studies in Portugal and Spain used exhaustive sampling in the included prison. Sampling method for the other studies was not reported. An extremely high prevalence was found in Norway (41.00%). However, this estimate was from a single high risk of selection bias study with a small sample size (*n* = 62). There is no subgroup analysis among prisoners reported.

#### PWID

An estimate of anti-HCV prevalence was found for 13 of the 34 countries in our review, ranging from 7.90 to 82.00% by country (Table 4). More than one estimate was available for 4 of 13 countries covered, with the most estimates for the UK (*n* = 3). Nine low risk of selection bias estimates from 6 countries (Croatia, Hungary, Germany, France, Spain and the UK) were available. Multiple low risk of selection bias estimates were available for a pooled estimate in the UK (*n* = 3) and France (*n* = 2). A high anti-HCV prevalence was found in Sweden (82.00%) and Spain (72.00%). A relatively low prevalence was reported in the UK (36.50%) and Croatia (34.04%). However, the estimate in Spain was of high risk of selection bias (score = 0). One article in the UK covered the vulnerable population in London, including both PWID and prisoners. Based on the results of quality assessment (score = 1), this result was also pooled into the estimate. Another article in the UK reported only a subgroup prevalence based on the years when the subjects were born. However, the exact estimate of subjects who were born in the early 1990s was not available. Only estimates from the subjects born after 2000 were pooled. The article in Croatia reported separated prevalence among PWID in the cities of Zagreb, Split and Rijeka. Data from the 3 cities in this article were pooled. The same situation came up in Germany with separated prevalence estimates in native German and former Soviet Union migrants, which were in the same article and pooled together. Subgroup analysis of age-specific prevalence was available for France [54] and Greece [56] and subgroup analysis of injection risk-specific prevalence was available for Greece [56], the UK [50], Hungary [58], and Spain [52]. Anti-HCV prevalence among PWID increased with increasing age in France (15.00% in 18–35 years and 56.00% in 35–65 years) [54] and Greece (52.60% in 15–24 years and 90.00% in older 65 years) [56]. Long-term injectors (those who had been injecting for more than 5 years) reported higher anti-HCV prevalence than new injectors in Greece (85.70% vs. 34.00%) [56], the UK (60.00% vs. 38.00%) [50] and Spain (77.10% vs. 59.40%) [52]. In Hungary [58], the anti-HCV prevalence in new psychoactive substances (NPS) injectors became the highest among the three injector groups (74.00% in NPS injectors, 59.00% in amphetamine injectors and 55.00% in opioid injectors).

**Table 4:** Summary of results from studies reporting anti-HCV prevalence in PWID

Author, year	Country	Recruiting period	Population as reported	Age, mean (SD)	Sample size	Anti-HCV prevalence (%) (95% CI)	Risk of selection bias
Aisyah, 2018 [48]	UK	2011–2013	Vulnerable populations in London	–	1207	11.40 (–)	Low risk
Hope, 2016 [49]	UK	1992–2013	Image and performance-enhancing drugs injectors in England and Wales	–	343	7.90 (–)	Low risk
Hope, 2015 [50]	UK	Since 1990	PWID from needle and syringe, opiate substitution treatment except for Scotland	–	123	41.70 (–)	Low risk
Valencia, 2018 [51]	Spain	January 2013–December 2016	PWUD who actively consumed heroin and/or cocaine, either smoked or injected	41.3(8.50)	946	33.30 (–)	High risk
Folch, 2016 [52]	Spain	2010–2011	PWID in harm reduction centres in Catalonia	–	754	72.00 (68.8–75.2)	Low risk
Leon, 2016 [53]	France	2004 and 2011	IDU	–	1242	43.40 (39.00–47.90)	Low risk
Weill-Barillet, 2016 [54]	France	2011	Drug users having injected or snorted drugs at least once in their life	39.0(−)	960	64.00 (59.20–68.20)	Low risk
Sypasa, 2017 [55]	Greece	2012–2013	During an HIV outbreak among PWID in Athens	–	431	49.90 (45.00–54.70)	High risk
Sheka, 2014 [56]	Greece	January 1997–December 2007	Intravenous drug users who attended the Greek Organisation against Drugs	–	2668	72.20 (–)	High risk
Derks, 2018 [57]	Germany	2011–2014	Current injectors in 8 German cities	–	1318	64.60 (–)	Low risk
Tarjan, 2017 [58]	Hungary	2011 and 2014	PWID injecting in the last month and attending SEPs or drug treatment centres	–	365	65.00 (–)	Low risk
Handanagic, 2016 [59]	Croatia	November 2014–February 2015	PWID in the cities of Zagreb, Split and Rijeka	–	399	38.30 (31.40–44.30)	Low risk
Kaberg, 2017 [60]	Sweden	7 April 2013–16 October 2014	PWID in the Stockholm needle exchange program (NEP)	39.3(1)	1386	82.00 (–)	High risk
Keegan, 2017 [61]	Ireland	31 January 2015	Patients attending agonist opioid treatment in a clinic in Dublin	50.2(18.3)	228	63.60 (–)	High risk
Skocibusic, 2016 [62]	Bosnia and Herzegovina	–	PWID of both sexes included in opiate substitution treatment in the southern part of Bosnia and Herzegovina	–	120	52.50 (–)	High risk
Svendsen, 2017 [63]	Norway	September 2015–November 2016	PWID in local opioid substitution clinic and day centres in Trondheim, Norway	–	304	41.00 (–)	High risk
Nosotti, 2016 [64]	Italy	–	IDU sample in Rome	–	261	41.70 (–)	High risk

### Comparison analysis

Pooled estimates by population and by country based on the results of quality assessment were shown in Table 5. Compared with the results reported in previous systematic reviews, our results updated the prevalence in Czech Republic, Poland, Portugal among the general population, Sweden and Spain among pregnant women, the Netherlands, the UK and France among MSM, France, Spain, Germany, Sweden and Bosnia and Herzegovina among PWID. Among the general population, the updated estimates were lower than previous ones in most available countries. A significant decrease in anti-HCV prevalence was shown in Italy (1.37% vs. 5.90%).

**Table 5:** Estimates of anti-HCV prevalence by population and by country

Country	Included references	Selected references	Sample size	Anti-HCV prevalence (%) (95% CI)	Baseline sample size	Baseline prevalence (%) (95% CI)
General population						
Spain	3	Single low risk	13678	0.8 (0.65–0.95) [20]	364	1.1(0.3–2.8) [17]
Italy	2	Pooled low risk	12444	1.37 (1.17–1.58) [22, 23]	4826	5.9(5.2–6.6) [17]
Poland	2	Single high risk	61805	1.5 (1.4–1.6) [24]	–	–
Czech Republic	1	Single low risk	3000	1.67 (1.21–2.13) [27]	–	–
Portugal	1	Single low risk	1627	0.54 (0.18–0.9) [28]	–	–
Netherlands	1	Single high risk	6036	0.56 (0.37–0.75) [33]	4046	0.1(0.0–0.2) [17]
Ireland	1	Single low risk	3795	0.98 (0.67–1.29) [26]	1478	0.1(0.0–0.4) [17]
Pregnant women						
UK	2	Single low risk	31467	0.1 (0.06–0.13) [31]	110621	1 [17]
Slovenia	1	Single low risk	31849	0.13 (0.09–0.17) [32]	90	4.4 [17]
Italy	1	Single high risk	5184	0.2 (0.08–0.32) [33]	10881	1.7 (1.4–1.9) [17]
Poland	1	Single low risk	38309	0.76 (0.67–0.85) [34]	1534	0.1 (0.0–0.3) [17]
Sweden	1	Single low risk	4112	0.27 (0.11–0.43) [35]	–	–
Spain	1	Single low risk	21379	0.55 (0.45–0.65) [36]	–	–
First-time blood donors						
Italy	1	Single low risk	1934612	0.09 (0.08–0.09) [37]	–	0.094 (0.085–0.104) [17]
Greece	1	Single high risk	3838919	0.03 (0.03–0.03) [38]	–	1.202 (1.114–1.295) [17]
MSM						
Netherlands	1	Single high risk	439	7.1 (4.69–9.51) [40]	–	–
UK	1	Single low risk	1140	1.8 (1.03–2.57) [39]	–	–
France	1	Single low risk	13051	5.1 (4.72–5.48) [41]	–	–
Prisoner						
UK	2	Pooled high risk	1484	7.9 (6.53–9.28) [42, 43]	5450	17.7(16.4–18.4) [18]
Portugal	2	Pooled high risk	82	16.51 (8.42–24.6) [44, 45]	151	34.4(26.9–42.6) [18]
Norway	1	Single high risk	62	51.6 (39.06–64.14) [63]		
Spain	1	Single high risk	436	16 (12.55–19.45) [47]	–	22.7(18.3–27.1) [18]
PWID						
UK	3	Pooled low risk	1818	36.5 (34.29–38.72) [48–50]	3144	49.1 (47.4–50.9) [18]
France	2	Pooled low risk	3015	57.26 (55.49–59.02) [53, 54]	–	–
Spain	2	Single low risk	754	72 (68.79–75.21) [52]	–	–
Greece	2	Pooled high risk	3099	69.67 (68.05–71.29) [55, 56]	1309	68.1 (65.5–70.6) [18]
Germany	1	Pooled low risk from a single study	1526	66.18 (63.8–68.55) [57]	–	–
Italy	1	Single high risk	261	47.1 (41.03–53.17) [64]	743	60.5 (56.8–64.0) [18]
Hungary	1	Single low risk	755	48.24 (44.67–51.81) [58]	652	24.1 (20.8–27.6) [18]
Croatia	1	Pooled low risk from a single study	830	34.04 (30.81–37.26) [59]	200	44 (37.0–51.2) [18]
Sweden	1	Single high risk	1386	82 (79.98–84.02) [60]	–	–
Ireland	1	Single high risk	228	63.6 (57.34–69.86) [61]	200	41.5 (34.6–48.7) [18]
Bosnia and Herzegovina	1	Single high risk	120	52.5 (43.53–61.47) [62]	–	–
Norway	1	Single high risk	304	41 (35.46–46.54) [63]	6342	63.0 (61.8–64.2)

Except for the Netherlands (0.56% vs. 0.10%) and Ireland (0.98% vs. 0.10%), prevalence increased. The same results were reported among high-risk populations, where prevalence in most countries decreased, except for the estimates among PWID in Hungary (48.24% vs. 24.10%).

## Discussion

This review is the first one to integrate and contrast prevalence estimates across the general population and 3 key high-risk groups in the EU/EEA. This review describes the finding of 46 publications estimating anti-HCV prevalence from 17 of the 34 EU/EEA countries, with 11 publications focusing on the general population, 7 on pregnant women, 2 on first-time blood donors, 3 on MSM, 6 on prisoners and 17 on PWID. In total, 48 estimates for anti-HCV prevalence were included, with 11 estimates of the general population, 7 estimates of pregnant women, 2 estimates of first-time blood donors, 5 estimates of MSM, 6 estimates of prisoners and 17 estimates of PWID. The anti-HCV prevalence varies widely between countries and populations.

For the majority of countries, the availability of estimates on the anti-HCV prevalence is limited. The estimates were reported in half of EU/EEA countries (17 of the 34). The results of quality assessment show there are potentially high risk of selection bias in half of available estimates (24 of the 48). The lack of low risk of selection bias prevalence estimates makes it challenging to gain an overview of the current epidemiological burden in the EU/EEA, especially in two high-risk groups, MSM and prisoners, with only 5 and 6 estimates included. Out of the stigma and reputational concerns, the access to MSM population is limited. The participation rate of screening in prison was high; however, the use of self-reported behavioral data and low sensitivity and specificity of laboratory test resulted in the limited inclusion of studies in prison environment.

This review confirms the diversity in anti-HCV prevalence among different populations. Compared with the prevalence among the general population, prevalence among first-time blood donors and pregnant women were found to be considerably lower, which agrees with the previous result that they are not a reliable proxy population to estimate prevalence in the general population. The prevalence among MSM, prisoners and PWID were found to be much higher than the corresponding prevalence in the general population. Of the high-risk groups considered, PWID reported the highest prevalence.

In contrast with most published systematic reviews, this study updates and adds new estimates of anti-HCV prevalence for three countries in the general population (Czech Republic, Poland and Portugal), for 2 countries in pregnant women (Spain and Sweden), for one country in MSM (the Netherlands), for one country in prisoners (Norway) and for 5 countries in PWID (Bosnia and Herzegovina, France, Germany, Spain and Sweden). Compared with previous estimates, the current estimates on prevalence among both the general and high-risk populations decreased in most available countries. While, the estimates of prevalence in the Netherlands [29] and Ireland [26] among the general population and in Hungary among PWID [58] increased. These estimates were all from single studies, and there may be a potential risk of selection bias. The estimate in the Netherlands was from a cohort consisting of more than 45-year-old elderly participants (mean age: 69.5 years) [29], which may lead to the relatively higher estimates than previous study (age of range: 15–44 years) [65]. The estimate in Ireland was from individuals whose specimens were submitted to the National Virus Reference Laboratory for diagnostic purpose, antenatal screening and pre-employment screening with most specimens being identified in older adults [26]. This was not likely to be completely representative of the general adult population and consequently led to higher estimates of prevalence. The estimate in Hungary was conducted among PWID attending drug treatment centres between 2011 and 2015 [58]. Since 2011, new psychoactive substances (NPS) have largely replaced traditionally injected drugs in Hungary [66]. NPS are unregulated products and have been associated with increases in injecting risks and HCV infection [66, 67]. High-risk environments for PWID may have played an important role in the increase of HCV prevalence in Hungary, especially for NPS injectors [68, 69]. Another systematic review [70] reported the anti-HCV prevalence for the 20 WHO European Region countries outside the EU/EEA. The anti-HCV prevalence outside the EU/EEA was higher than inside the EU/EEA among the general population (0.50–13.00% vs. 0.54 to 1.50%), and first-time blood donors (0.03–6.40% vs. 0.03–0.09%), but lower among PWID (5.30–73.00% vs. 7.90–82.00%).

The strength of this review is that it covers all the general population and high-risk groups. The previous reviews, due to pragmatic reasons, extracted prevalence estimates for PWID from the data repository from the ECDDA. It is possible that this data set is not exhaustive. In our review update, however, PWID-specific search terms were used to identify potential studies. We believe that the description provided gives a sufficiently thorough review of recently published anti-HCV prevalence estimates. With the latest prevalence estimates, this study can contribute to the analyses in cost of illness, cost-effectiveness and budget impact to optimise the healthcare resource utilisation in hepatitis C management and a HCV eradication program in Europe. The fundamental approaches to control a HCV epidemic are prevention of new infections and management of existing infections. Currently, there is no vaccine for the prevention of the HCV infection. A needle/syringe provision (NSP) and opioid substitution treatment (OST) as the main primary prevention strategy and HCV antiviral treatment as a treatment-as-prevention strategy are the key components to reduce HCV prevalence. Several studies have shown that high coverage of NSP and OST [71–74] and scaling up HCV treatment, especially treatment with DAAs that possess high efficacy and improved safety profiles [11, 13, 14, 75], can lead to substantial reductions in anti-HCV prevalence. The use of DAAs could make hepatitis C a rare disease in the next 20 to 30 years [76]. Despite advances in prevention and treatment, the HCV related disease burden is expected to increase before it starts declining, as well as corresponding healthcare cost [77]. This highlights the importance of optimisation of resource allocation in HCV eradication program.

However, this study confirmed that there was an evidence gap on anti-HCV prevalence among lots of EU/EEA countries. This limitation of this study also provides the idea for further research. In some countries, no national studies had been reported, thus local and regional data were assumed to be reflective of the whole country. However, by assessing the methodological quality of the studies, this limitation can be further mitigated.

## Conclusion

This review emphasises the heterogeneity in anti-HCV prevalence across different population groups in Europe. The prevalence also shows considerable diversity between EU/EEA countries. There are many countries that are not described in our results, emphasising the existing need to develop robust epidemiological studies.

## Additional file

Additional file 1: Methodological details of study. 1.1 Search strategy for studies in Medline and Embase via Ovid; 1.2 Inclusion and exclusion criteria; 1.3 Results of quality assessment for risk of selection bias. (DOCX 95 kb)

## Data Availability

The full search strategy, the inclusion/exclusion criteria and results on quality assessment of included studies are available in the Additional file 1.

## Abbreviations

EMCDDA: European Monitoring Centre for Drugs and Drug Addiction
EU/EEA: European Union/European Economic Area
HCV: Hepatitis C Virus
HIV: Human Immuno-deficiency Virus
MSM: Men Who Have Sex with Men
NPS: New Psychoactive Substance
NSP: Needle/syringe Provision
OST: Opioid Substitution Treatment
PRISMA: Preferred Reporting Items for Systematic Reviews and Meta-Analyses
PWID: People Who Inject Drugs
UK: United Kingdom
