# Supplementary material for: Prevalence of hepatitis C infection among the general population and high-risk groups in the EU/EEA: a systematic review update
Source: BMC Infect Dis. 2019 Jul 23;19:655. doi: 10.1186/s12879-019-4284-9 (PMC6647266; doi:10.1186/s12879-019-4284-9)
Supplement: Supplementary file 1 — Methodological details of study. 1.1 Search strategy for studies in Medline and Embase via Ovid; 1.2 Inclusion and exclusion criteria; 1.3 Results of quality assessment for risk of selection bias. (DOCX 95 kb) [file 12879_2019_4284_MOESM1_ESM.docx]

# Additional file 1.1. Search strategy for studies in Medline and Embase via Ovid

| ID | Category | Search terms | Hits |
| --- | --- | --- | --- |
| #1 | Population | exp hepatitis C/ or (Hepatitis C virus or Hepatitis C viruses).mp. or HCV.mp. or hepaciviruses.mp. or (Hepatitis C-Like Viruses or Hepatitis C-Like Virus or Hepatitis C Like Viruses).mp. | 232,548 |
| #2 |  | (pwid or idu or injecting drug users or intravenous drug users or substance abuse* or drug abuse* or drug users).tw. | 8,501,302 |
| #3 | Epidemiology | prevalence.tw. or incidence.tw. or epidemiolo$.tw. or *Prevalence/ or *Incidence/ or *Epidemiology/ or seroepidemiological studies/ or seroepidemiological studies/ or anti-hcv or anti hcv or hcv antigens ir hcv antibodies or hcv rna or carrier* or serologic* markers | 3,950,524 |
| #4 | Geographic scope | (((((United kingdom or Britain or British or English or Scotland or Scottish or Wales or Welsh or Northen Ireland or London or East midlands or West midlands or Yorkshire or East Anglia or Bedfordshire or Hertfordshire or Essex or Peterborough or Cambridgeshire or Norfolk or Suffolk or Luton or Bedford or Southend on sea or Thurrock or Derbyshire or nottinghamshire or Leicestershire or Rutland or Northamptonshire or Lincolnshire or Derby or Leicester or Northamptonshire or nottingham or Northumberland).tw. or Tyne.mp.) and Wear.tw.) or Tees Valley.tw. or Durham.tw. or Darlington.tw. or Hartlepool.tw. or Stockton on tees.tw. or Northumberland.tw. or Teesside.tw. or Sunderland.tw. or Tyneside.tw. or Cumbria.tw. or Cheshire.tw. or Manchester.tw. or Lancashire.tw. or Merseyside.tw. or (Blackburn and Darwen).tw. or Blackpool.tw. or Chester.tw. or Liverpool.tw. or Sefton.tw. or Warrington.tw. or Wirral.tw. or Berkshire.tw. or Buckinghamshire.tw. or Oxfordshire.tw. or Hampshire.tw. or Isle of Wight.tw. or Kent.tw. or Surrey.tw. or Sussex.tw. or (Brighton and Hove).tw. or Medway.tw. or Milton keynes.tw. or Portsmouth.tw. or Southampton.tw. or Devon.tw. or Dorset.tw. or Somerset.tw. or Gloucestershire.tw. or Wiltshire.tw. or Bristol.tw. or Bath.tw. or Bournemouth.tw. or Poole.tw. or Bristol.tw. or Plymouth.tw. or Swindon.tw. or Torbay.tw. or Herefordshire.tw. or Worcestershire.tw. or Warwickshire.tw. or Shropshire.tw. or Staffordshire.tw. or Birmingham.tw. or Coventry.tw. or Dudley.tw. or Sandwell.tw. or Shropshire.tw. or Solihull.tw. or stoke on trent.tw. or Telford.tw. or Wrekin.tw. or Walsall.tw. or Warwickshire.tw. or Wolverhampton.tw. or Worcestershire.tw. or Barnsley.tw. or Doncaster.tw. or Rotherham.tw. or Bradford.tw. or Calderdale.tw. or Kirklees.tw. or Kingston.tw. or Leeds.tw. or Sheffield.tw. or Wakefield.tw. or Antrim.tw. or Ards.tw. or Armagh.tw. or Ballymena.tw. or Ballymoney.tw. or Banbridge.tw. or Carrickfergus.tw. or Castlereagh.tw. or Coleraine.tw. or Cookstown.tw. or Craigavon.tw. or Derry.tw. or (Down and (district or council)).tw. or Fermanagh.tw. or Dungannon.tw. or Fermanagh.tw. or Larne.tw. or Limavady.tw. or Lisburn.tw. or Magherafelt.tw. or Moyle.tw. or (Newry and Mourne).tw. or Newtownabbey.tw. or Omagh.tw. or Strabane.tw. or Londonderry.tw. or Tyrone.tw. or Belfast.tw. or Aberdeen.tw. or Aberdeenshire.tw. or Angus.tw. or Dundee.tw. or (Argyll and bute).tw. or Clackmannanshire.tw. or Fife.tw. or Ayrshire.tw. or Dunbartonshire.tw. or Lothian.tw. or Renfrewshire.tw. or Edinburgh.tw. or Falkirk.tw. or Glasgow.tw. or Highland*.tw. or Inverclyde.tw. or Midlothian.tw. or Moray.tw. or Lanarkshire.tw. or (Perth and Kinross).tw. or Stirling.tw. or orkney Islands.tw. or Eileanan Siar.tw. or Shetland Islands.tw. or Bridgend.tw. or Neath Port Talbot.tw. or Cardiff.tw. or (Vale and Glamorgan).tw. or Central Valleys.tw. or Conwy.tw. or Denbighshire.tw. or Flintshire.tw. or Wrexham.tw. or Gwent Valleys.tw. or Gwynedd.tw. or (Isle and Anglesey).tw. or Monmouthshire.tw. or Newport.tw. or Powys.tw. or Swansea.tw. or Ceredigion.tw. or Carmarthenshire.tw. or Pembrokeshire.tw. or Merthyr Tydfil.tw. or Rhondda Cynon Taff.tw. or Blaenau Gwent.tw. or Caerphilly.tw. or Torfaen.tw. or Caithness.tw. or Sutherland.mp.) and Ross.tw.) or Cromarty.tw. or Teeside.tw. or Tyneside.tw. or Wearside.tw. or West Mercia.tw. or Avon.tw. or Ulster.tw. or Derry.tw. or Medway.tw. or East Riding.tw. or West Riding.tw. or Lake District.tw. or Peak District.tw. or Cumberland.tw. or Dartmoor.tw. or Exmoor.tw. [mp=ti, ab, hw, tn, ot, dm, mf, dv, kw, fx, dq, nm, kf, px, rx, an, ui, sy] | 6,225 |
| #5 |  | (Sweden or Sverige or Swedish or Svenska or Stockholm* or Norrland or Svealand or Mellansverige or Smaland or Sydsverige or Vastsverige or orebro or Ostergotland* or Vastergotland* or Skara* or Bohus* or Dalsland or Narke or Sodermanland or Uppsala or Uppland or Vastmanland* or Jamtland* or Harjedalen or Vasternorrland* or Dalarna or Kopparberg or Gavleborg* or Gastrikland or Halsingland or Varmland* or Gotland* or Oland or Jonkoping* or Kalmar* or Kronoberg* or Blekinge or Skane* or Norrbotten* or Vasterbotten* or Lappland or Angermanland or Medelpad or Halland* or Gotaland* or Gothenburg or Goteborg* or Malmo* or Vasteras or Linkoping or Helsingborg or Halsingborg or Norrkoping or Gavle or Umea or Lulea or Karlstad or Kalmar or Huddinge or Solna or Ostersjo* or Malaren* or Malardalen).tw. | 169,938 |
| #6 |  | (((Spain or Espana or Spanish or Espanol* or Spaniard* or Madrid or andalucia or andalusia or Aragon or Cantabria or Canarias or Canary Islands).tw. or Castile.mp.) and leon.tw.) or Castilla y Leon.tw. or Castile La Mancha.tw. or Castilla La Mancha.tw. or Cataluna.tw. or Catalonia.tw. or Ceuta.tw. or Melilla.tw. or Navarra.tw. or Navarre.tw. or Valencia.tw. or Valencian.tw. or Extremadura.tw. or Galicia.tw. or Balears.tw. or Balearic Islands.tw. or Baleares.tw. or La Rioja.tw. or Pais Vasco.tw. or Basque Country.tw. or Asturias.tw. or Murcia.tw. or Coruna.tw. or Alava.tw. or Araba.tw. or Albacete.tw. or Alicante.tw. or Alacant.tw. or Almeria.tw. or Asturias.tw. or Avila.tw. or Badajoz.tw. or Badajos.tw. or Barcelona.tw. or Burgos.tw. or Caceres.tw. or Cadiz.tw. or Castellon.tw. or Castello.tw. or Ciudad Real.tw. or Cuenca.tw. or Eivissa.tw. or Ibiza.tw. or Formentera.tw. or El Hierro.tw. or Fuerteventura.tw. or Girona.tw. or Gerona.tw. or Gran Canaria.tw. or Granada.tw. or (Guadalajara not Mexic*).tw. or Guipuzcoa.tw. or Gipuzkoa.tw. or Huelva.tw. or Huesca.tw. or Jaen.tw. or La Gomera.tw. or La Palma.tw. or Lanzarote.tw. or Leon.tw. or Lleida.tw. or Lerida.tw. or Lugo.tw. or Malaga.tw. or Mallorca.tw. or Majorca.tw. or Menorca.tw. or Minorca.tw. or Murcia.tw. or Ourense.tw. or orense.tw. or Palencia.tw. or Pontevedra.tw. or Salamanca.tw. or Segovia.tw. or Sevilla.tw. or Seville.tw. or Soria.tw. or Tarragona.tw. or Tenerife.tw. or Teruel.tw. or Toledo.tw. or Valencia.tw. or Valladolid.tw. or Vizcaya.tw. or Biscay.tw. or Zamora.tw. or Zaragoza.tw. or Saragossa.tw. or Bilbao.tw. or Bilbo.tw. or Compostela.tw. or San Sebastian.tw. or Donostia.tw. or Vitoria.tw. or Oviedo.tw. or Pamplona.tw. or Logrono.tw. or Gasteiz.tw. [mp=ti, ab, hw, tn, ot, dm, mf, dv, kw, fx, dq, nm, kf, px, rx, an, ui, sy] | 74,246 |
| #7 |  | (Slovenia* or Slovenija or slovensk* or Ljubljana or Gorenjska or Carniola or Goriska or Gorizia or Koroska or Carinthia or notranjsko kraska or Obalno kraska or Coastal krast or Podravska or Pomurska or Savinjska or Spodnjeposavska or Zasavska or Osrednjeslovenska or Maribor or Celje or Kranj or Velenje or Koper or Capodistria or Novo mesto or Ptuj or Trbovlje or Kamnik or Murska or Sobota or Nova Gorica).tw. | 10,773 |
| #8 |  | (Slovakia or Slovensk* or Slovak* or Bratislav* or Trnav* or Trnava or Nitrian* or Nitra or Trencian* or Trencin or Banskobystri* or Banska Bystrica or Zilina or Zilin* or Trnava or Trnav* or Presov or Presov* or Kosic* or (Martin and (city or Svaty)) or Poprad).tw. | 16,116 |
| #9 |  | (Romania or Rumania or Roumania or Romanian or Roman or Bucharest or Bucuresti or Alba or Brasov or Covasna or Harghita or Mures or Sibiu or Bacau or Botosani or Iasi or Neamt or Suceava or Vaslui or Bihor or Bistrita Nasaud or Cluj or Maramures or Salaj or Satu Mare or Arges or Calarasi or Dambovita or Giurgiu or Ialomita or Prahova or Teleorman or Braila or Buzau or Constanta or Galati or Tulcea or Vrancea or Dolj or Gorj or Mehedinti or (Olt and (river or county or region or judetul or Raul)) or Valcea or Vilcea or Arad or Caras-Severin or Hunedoara or Timis or Ilfov or Timisoara or Constanta or Craiova or Ploiesti or oradea or Cluj-Napoca or Deva).tw. | 41,596 |
| #10 |  | (Portugal or Portugues* or Lisboa or Lisbon or Leira or Santarem or Beja or Faro or Evora or Portalegre or Castelo Branco or Guarda or Aveiro or Viseu or Braganca or Vila real or Viana do Castelo or Alentejo or Azores or Acores or Madeira or Os Montes or (Ave and (community or intermunicipal or comunidade)) or Mondego or Vouga or Beira or Cavado or Lafoes or Douro or Porto or Tejo or Minho or Setubal or Pinhal or Serra da Estrela or Tamega or Algarve or Gaia or Amadora or Braga or (Agualva and Cacem) or Funchal or Coimbra or Almada).tw. | 62,975 |
| #11 |  | (Poland or Polska or Polish or Polski or Pole or Poles or Polak or Polka or Polacy or Polacy or Warsaw or Warszawa or Wielkopolskie or Pomerania* or Pomorskie or Kuyavian or Kujawsko or Malopolskie or Lodz or Lodzkie or Silesia* or Slask or Dolnoslaskie or Lublin or Lubelskie or Lubus or Lubusz or Lubuskie or Masovia or Mazowske or Masovian or Mazowieckie or Opole or Opolskie or Podlaskie or Podlachia or Podlasie or Subcarpathian* or Carpathian* or Podkarpackie or Swietokrzyskie or Slaskie or Slask or Varmia Mazuria or Varmian Mazurian or Varmia Masuria or Varmian Masurian or Warmia Mazury or Warminsko Mazurskie or Zachodniopomorskie or Krakow or Cracow or Wroclaw or Poznan or Gdansk or Szczecin or Bydgoszcz or Katowice or Bialystok or Olsztyn or Kielce or Zielona Gora or Torun or Gorzow Wielkopolski).tw. | 179,308 |
| #12 |  | (Netherlands or Nederland* or Dutch* or Amsterdam or Drenthe or Flevoland or Friesland or Fryslan or Gelderland or Guelders or Groningen or Limburg or North Brabant or Noord Brabant or Holland or Overijssel or Overissel or Utrecht or Zeeland or Rotterdam or Hague or Eindhoven or Tilburg or Almere or Breda or Nijmegen or Nimeguen).tw. or ((Malta or Maltese or Valletta or Gozo or Ghawdex).tw. or Malta.oi. or Maltese.oi. or Valletta.oi. or Gozo.oi. or Ghawdex.oi.) or ((Luxembourg* or Luxemburg or Letzebuerg or Diekirch or Grevenmacher).tw. or Luxembourg*.oi. or Luxemburg.oi. or Letzebuerg.oi. or Diekirch.oi. or Grevenmacher.oi.) or (Lithuania* or Lietuvos Respublika or Lietuva or lietuviu or Vilnius or Vilniaus or Kaunas or Kauno or Klaipeda or Klaipedos or Panevezys or Panevezio or Siauliai or Siauliu or Alytus or Alytaus or Taurages or Taurage or Marijampoles or Marijampole or Telsiu or Telsiai or Utenos or Utena or Mazeikiai or Jonava or Mazeikiu or Jonavos).tw. | 212,473 |
| #13 |  | (Latvi* or Latvija* or Riga or Courland or Kurzeme or Kurland or Latgale or Lettgallia or Latgola or Vidzeme or Vidumo or Semigallia or Semigalia or Zemgale or Pieriga or Daugavpils or Dinaburg or Liepaja or Libau or Jelgava or Jurmala or Jekabpils or Jakobstadt or Rezekne or Rezne or Rositten or Valmiera or Wolmar or Ventspils or Windau).tw. | 4,228 |
| #14 |  | (Italy or Italia* or Rome or Roma or Abruzzo or Abruzzi or Basilicata or Lucania or Calabria or Campania or Emilia Romagna or friuli venezia giulia or Lazio or Latium or Liguria* or Lombardy or Lombardia or Marche or Marches or Molisano or Molise or Piedmont* or Piemonte or Sardinia or Sardegna or Sicily or Sicilia or Toscana or Tuscany or Trentino or Trento or Umbria or Veneto or Triveneto or Puglia or Apulia or Bolzano or Bozen or Milan or Milano or Naples or Napoli or Turin or Torino or Palermo or Genoa or Genova or Bologna or Florence or Firenze or Bari or Catania or Venezia or Venice or Padova or Padua or Siena or Bologna or Trieste or Urbino or Aosta or Aoste or Perugia or Brescia or Cagliari or Catanzaro or L Aquila or Ancona).tw. | 296,523 |
| #15 |  | (Ireland or Eire or Irish* or Dublin or Fingal or Dun Laoghaire or Wicklow or Wexford or Carlow or Kildare or Meath or Louth or Monaghan or Cavan or Longford or Westmeath or Offaly or Laois or Kilkenny or Waterford or Cork or Kerry or Limerick or Tipperary or Clare or Galway or Mayo or Roscommon or Sligo or Leitrim or Donegal or Drogheda or Dundalk or Swords or Bray or Navan or Munster or Leinster or Connacht).tw. | 238,499 |
| #16 |  | (Hungar* or Budapest or Transdanubia or Magyarorszag or magyar or Dunantuli or Dunantul or Great Plain or Alfold es eszak or Eszak Alfold or Del Alfold or Bacs or Kiskun or Northen Alfold or Sourthen Alfold or Baranya or Bekes or borsod or Abauj or Zemplen or Fovaros or Csongrad or Fejer or gyor or moson or sopron or hajdu or bihar or Heves or jasz nagykun szolnok or komarom or esztergom or Nograd or Pest or Somogy or szabolcs or szatmar or bereg or Tolna or Vas or Veszprem or Zala or Zalaegerszeg or Debrecen or Miskolc or Szeged or Pecs or Gyor or Nyiregyhaza or Kecskemet or Szekesfehervar or Szombathely or Bekescsaba or Eger or Tatabanya or Salgotarjan or Kaposvar or Szekszard).tw. | 191,712 |
| #17 |  | (Greece or Hellenic republic or Greek* or Ellada or Elliniki Dimokratia or Hellas or Hellenes or Attica or Attiki or Makedonia or Macedonia or Thraki or Thrace or Crete or Kriti or Epirus or Ipeiros or Ionia Nisia or Ionion neson or Ionian islands or North aegean or Aegean islands or Nisoi Agaiou or notio Aigaio or Peloponnese or Peloponnisos or Voreio Aigaio or South aegean or Thessaly or Thessalia or Cycklades or Kiklades or Dodecanese or Dodekanisa or Mount athos or Omicronros Alphathos or Athens or Athina or Thessaloniki or Thessalonica or Patras or Patra or Heraklion or Heraclion or Iraklion or Irakleion or Iraklio or Larissa or Larisa or Volos or Rhodes or Rodos or Ioannina or Janina or Yannena or Chania or Chalcis or Chalkida or Alexandroupoli).tw. | 62,048 |
| #18 |  | (German* or Deutschland or Deutsch* or Bundesrepublik or Westdeutschland or Ostdeutschland or Baden or Wuerttemberg or Wurttemberg or Bayern or Bavaria or Berlin or Brandenburg or Bremen or Hamburg or Hessen or Hesse or Hessia or Mecklenburg or Vorpommern or Pomerania or Niedersachsen or Neddersassen or Saxony or Niederbayern or North Rhine or Westphalia or Westfalen or Rhineland Palatinate or Rheinland Pfalz or Saarland or Sachsen or Schleswig Holstein or Thuringia or Thuringen or Thueringen or Freiburg or Karlsruhe or Calsruhe or Stuttgart or Tubingen or Oberbayern or Upper palatinate or Oberpfalz or Franken or Franconia or Oberfranken or Mittelfranken or Schwaben or Unterfranken or Swabia or Darmstadt or Giessen or Giessen or Kassel or Arnsberg or Cologne or Koln or Koeln or Detmold or Dusseldorf or Duesseldorf or Munster or Muenster or Munich or Munchen or Muenchen or Frankfurt or Dortmund or Essen or Nurnberg or Nuernberg or Nuremberg or Hanover or Hannover or Leipzig or Dresden or Ruhrgebiet or Revier or Ruhrpott or Pott or Ruhr).tw. | 475,543 |
| #19 |  | (France or French* or Francais or Alsace or Aquitaine or Auvergne or Brittany or Bretagne or Bourgogne or Burgundy or Champagne Ardenne or Franche Comte or Ile de France).tw. | 279,987 |
| #20 |  | (Finland or Finnish* or Suomi* or Lapland or Lappi or Lappland or Ostrobothnia or Pohjanmaa or Osterbotten or Kainuu or Kajanaland* or Karelia or Karjala or Karelen or Savonia or Savo or Savolax or Pirkanmaa or Birkaland or Satakunta or Satakunda or Tavastia or Tavastland or Paijat Hame or Kanta Hame or Uusimaa or Nyland or Kymenlaakso or Kymmenedalen or Aland or Ahvenanmaa or Helsinki or Helsingfors or Espoo or Esbo or Tampere or Tammerfors or Vantaa or Vanda or Oulu or Uleaborg or Turku or Abo or Jyvaskyla or Kuopio or Lathi or Lahtis or Kouvola).tw. | 114,851 |
| #21 |  | (Estonia* or Eesti or Esti or Tallinn or Harju or Harjumaa or Hiiu or Hiiumaa or Ida Viru or Ida Virumaa or Jarvamaa or Jarva or Jogevamaa or Jogeva or Laanemma or Laane or Parnu or Parnumaa or Polva or Polvamaa or Rapla or Raplamaa or Saare or Saaremaa or Tartu or Tartumaa or Valga or Valgamaa or Valgamaakond or Viljandimaa or Viljandi or Voru or Vorumaa or Narva or Parnu or Kohtla Jarve or Viljandi or Rakvere or Maardu or Sillamae or Kuressaare).tw. | 8,296 |
| #22 |  | (Denmark or Danish* or Danmark or dansk* or Hovedstaden or Midtjylland or Nordjylland or Sjaelland or Sealand or Syddanmark or Jutland or Jylland or Nordjylland or Sonderjyllands or Zealand region or region Zealand or Hillerod or Viborg or Aalborg or Alborg or Soro or Vejle or Copenhagen or Kobenhavn or Arhus or Aarhus or Roskilde or Odense or Frederiksberg or Esbjerg or Gentofte or Gladsaxe or Randers or Kolding).tw. | 114,979 |
| #23 |  | (Czech* or Cesk* or Stredoces* or Jihoces* or Bohemia or Bohemian region or Kralovehradec* or Hradec Kralove or Karlovars* or Karlovy Vary or Liberec* or Moravskoslezs* or Moravian Silesian or Olomouc* or Pardubic* or Plzen* or Pilsen or Prage or Praha or Prag or Jihomorav* or Moravia or Moravian or Morava or Usteck* or Usti or Vysocina or Zlin or Zlinsk* or Ceske Budejovice or Budweis or Brno or Ostrava).tw. | 46,740 |
| #24 |  | (Cyprus or Cypriot* or Kypros or Kibris or kypriaki* or Nicosia or Lefkosa or Lefkosia or Famagusta or Magusa or Ammochostos or Gazimagusa or Kyrenia or Girne or Keryneia or Larnaca or Larnaka or Limassol or Lemesos or Limasol or Leymosun or Paphos or Pafos or Baf or Gazibaf or Protaras or Pergamos or Beyarmudu or Morfou or Guzelyurt or Omorfo or Morphou or Aradippou).tw. | 7,850 |
| #25 |  | (Croatia* or Hrvatsk* or hrvat or Bjelovar or Bjelovarsko bilogorska or Brod Posavina or Brodsko posavska or Dubrovnik Neretva or dubrovacko neretvanska or Istria or Istarska or Karlovacka or Karlovac or Koprivnicko krizevacka or Koprivnica or Krizevci or Krapina Zagorje or Krapinsko zagorska or Lika Senj or Licko senjska or Medimurska or Medimurje or Osijek or Osjecko or Baranja or Osjecko baranjska or Pozega Slavonia or Pozesko slavonska or Primorje Gorski Kotar or Primorsko goranska or Sibensko kninska or Sibensko kninske or Sibenik or Knin or Sisak or Sisacko moslavacka or Moslavina or Splitsko dalmatinska or Split or Dalmatia or Varazdin or Varazdinska or Viroviticko-podravska or Virovitica or Podravina or Vukovarsko srijemska or Vukovar or Srijem or Zadar or Zadarska or Zagreb or Zagrebacka or Rijeka or Velika gorica or Slavonski brod or Pula).tw. | 142,473 |
| #26 |  | (Bulgaria* or Sofia or Gabrovo or Blagoevgrad or Pirin Macedonia or Burgas or Dobrich or Haskovo or Kardzhali or Kurdzhali or Kyustendil or Lovech or Montana or Pazardzhik or Pernik or Pleven or Plovdiv or Razgrad or Rousse or Ruse or Shumen or Sliven or Silistra or Smolyan or Stara Zagora or Targovishte or Varna or Tarnovo or Vidin or Vratsa or Vratza or Yambol).tw. | 24,069 |
| #27 |  | (Belgi* or Belge or Belgisch or Brussel* or Bruxelles or Bruxelloise or Flemish or Flamand or Flemisch or Flanders or Flandern or Flandre or Vlaanderen or Vlaams or Flamande or Waals or Walloon* or Wallon* or Antwerp* or Anvers or Ostflandern or Vlaams Brabant or Limbourg or Limburg or Hainault or Hainaut or Henegouwen or Hennegau or Liege or Luik or Luttich or Namur or Namen or Westflandern or Waals Brabant or Ghent or Gent or Gand or Charleroi or Bruges or Brugge or Schaerbeek or Schaarbeek or anderlecht or Leuven or Louvain or Hasselt or Mons or Wavre or Waver).tw. | 88,592 |
| #28 |  | (Austria* or Vienna or Wien or Osterreich* or Sudosterreich or Westosterreich or Niederosterreich or Burgenland or Carinthia or Karinthia or Karnten or Oberosterreich or Styria or Steiermark or Salzburg or Saizburg or Tyrol or Tirol or Becs or Vorarlberg or Bregenz or Linz or Eisenstadt or Innsbruck or Graz or Klagenfurt or Polten or Villach or Wels or Dornbirn or Feldkirch or Steyr).tw. | 59,663 |
| #29 |  | (Iceland or Icelandic* or islenska* or Icelander* or islendinga* or Reykjavik or Reykjavikurborg or Hofudborgarsvaedid or Sudurnes or Vesturland or Vestfirdir or Westfjords or Nordurland or Austurland or Sudurland or Kopavogur or Hafnarfjordur).tw. | 12,912 |
| #30 |  | (Switzerland or Schweiz or Schweizerische or Swiss or Suisse or Aargau or Argovia or Ausserrhoden or Outer Rhodes or Innerrhoden or Inner Rhodes or Basel or Bern or Berne or Fribourg or Freiburg or Geneva or Geneve or Glarus or Graubunden or Grisons or Grigioni or jura or Lucerne or Luzern or Neuchatel or Zurich or (Uri and (canton or Kanton)) or Schwyz or Obwalden or Nidwalden or Zug or Solothurn or Schaffhausen or Thurgau or Thurgovia or Ticino or Tessin or Vaud or Valais or Wallis or St Gallen or Lausanne or Winterthur or Winterthour or Lugano or Biel or Bienne).tw. | 261,584 |
| #31 |  | (((((Norway or Norwegian* or Norge or Noreg or Norgga or Ostfold or Akershus or Oslo or Hedmark or Oppland or Buskerud or Vestfold or Telemark or Aust Agder or Vest Agder or Rogaland or Hordaland or Sogn og fjordane).tw. or Sogn.mp.) and fjordane.tw.) or sogn fjordane.tw. or More og Romsdal.tw. or More.mp.) and Romsdal.tw.) or More Romsdal.tw. or Trondelag.tw. or Nordland.tw. or Troms.tw. or Finnmark.tw. or Bergen.tw. or Stavanger.tw. or Sandnes.tw. or Trondheim.tw. or Kristiansand.tw. or Drammen.tw. or Fredrikstad.tw. or Sarpsborg.tw. or Porsgrunn.tw. or Skien.tw. or Tonsberg.tw. or Alesund.tw. [mp=ti, ab, hw, tn, ot, dm, mf, dv, kw, fx, dq, nm, kf, px, rx, an, ui, sy] | 7,123 |
| #32 |  | (Liechtenstein or Vaduz or Triesenberg or Triesen or Schellenberg or Schaan or Ruggell or Planken or Mauren or Gamprin or Eschen or Balzers).tw. | 782 |
| #33 |  | (European Union or Europe).mp. or Europa.tw. or Europe*.tw. or Scandinavia*.tw. or Scandinavia*.oi. or Mediterranean.tw. or EEA countries.tw. or EU country.tw. or EU countries.tw. or global*.tw. or world.tw. or worldwide.tw. [mp=ti, ab, hw, tn, ot, dm, mf, dv, kw, fx, dq, nm, kf, px, rx, an, ui, sy] | 2,497,591 |
| #34 |  | or/ 4-33 | 5,042,394 |
| #35 | Total 1 | 1 and 3 and 34 | 13,866 |
| #36 |  | Limit 35 to "year=2015-current" | 2,790 |
| #37 | Total 2 | 1 and 2 and 3 and 34 | 1,986 |
| #38 |  | Limit 37 to yr="2009-Current" | 1,081 |

# Additional file 1.2. Inclusion/exclusion criteria

| **Criteria elements** | **Inclusion criteria** | **Exclusion criteria** |
| --- | --- | --- |
| **Population** | General population, pregnant women, blood donor, PWID, MSM, prisoners | Articles reporting data on non-representative populations/specific high-risk groups only, e.g. homeless, dialysis patients etc. |
| **Outcomes of interest** | Anti-HCV prevalence with disease specific markers in humans and sex/age-stratified seroprevalence | Not specified serological markers Articles reporting data from a study non conducted in humans or study on diagnostic and/or laboratory methods |
| **Study designs** | Articles reporting original data | Articles reporting modelled or extrapolated data only Articles reporting only self-reported/unconfirmed prevalence Opinion papers, editorials, guidelines or recommendations, and systematic reviews or meta-analysis |
| **Publication timeframe** | Published after 2015 to present | None |
| **Publication type** | Full text, poster, abstracts | Abstracts and poster with full text published |
| **Geographical scope** | One or more EU/EEA MS and/or any of their regions/districts | Articles reporting data on non EU/EEA countries only |

# Additional file1.3. Results of quality assessment for risk of selection bias

| Author, year | Age | Gender | Sampling method | Geographical coverage | Total scores | Risk of selection bias |
| --- | --- | --- | --- | --- | --- | --- |
| General population |  |  |  |  |  |  |
| Viejo, 2018[48] | 1 | 1 | 0 | 0 | 2 | High risk |
| Lavin, 2017[49] | 1 | 1 | 1 | 2 | 5 | Low risk |
| Quesada, 2015[50] | 1 | 0 | 0 | 0 | 1 | High risk |
| Andriulli, 2018[19] | 1 | 1 | 2 | 2 | 6 | Low risk |
| Morisco, 2017[20] | 1 | 1 | 2 | 1 | 5 | Low risk |
| Walewska-Zielecka, 2017[21] | 1 | 1 | 0 | 0 | 2 | High risk |
| Clifford, 2017[51] | 1 | 0 | 0 | 0 | 1 | High risk |
| Garvey, 2017[34] | 1 | 1 | 0 | 2 | 4 | Low risk |
| Chlibek, 2017[52] | 1 | 1 | 0 | 1 | 3 | High risk |
| Carvalhana, 2016[53] | 1 | 1 | 2 | 2 | 6 | Low risk |
| Plompen, 2015[33] | 0 | 1 | 0 | 0 | 1 | High risk |
| Pregnant women |  |  |  |  |  |  |
| Orkin, 2016[22] | NR | NR | 0 | 1 | 1 | High risk |
| Cortina-Borja, 2016[23] | NR | NR | 2 | 1 | 3 | Low risk |
| Kopilovic, 2015[25] | NR | NR | 1 | 2 | 3 | Low risk |
| Lembo, 2017[54] | NR | NR | 0 | 0 | 0 | High risk |
| Walewska-Zielecka, 2016[24] | NR | NR | 0 | 2 | 2 | Low risk |
| Millbourn, 2017[55] | NR | NR | 2 | 1 | 3 | Low risk |
| Munoz-Gamez, 2016[56] | NR | NR | 0 | 2 | 2 | Low risk |
| Blood donor |  |  |  |  |  |  |
| Velati, 2018[57] | 1 | 1 | 2 | 2 | 6 | Low risk |
| Politis, 2018[58] | 0 | 0 | 0 | 2 | 2 | High risk |
| MSM |  |  |  |  |  |  |
| Ireland, 2017[26] | NR | NR | NR | 1 | 1 | Low risk |
| Vanhommerig, 2013[59] | NR | NR | NR | 0 | 0 | High risk |
| Cotte, 2018[27] | NR | NR | NR | 2 | 2 | Low risk |
| Prisoner |  |  |  |  |  |  |
| Ekeke, 2018[60] | 0 | 0 | 0 | 0 | 0 | High risk |
| Patel, 2016[61] | 0 | 0 | 0 | 0 | 0 | High risk |
| Casella, 2016[62] | 0 | 0 | 0 | 0 | 0 | High risk |
| Liberal, 2017[63] | 0 | 0 | 2 | 1 | 3 | High risk |
| Svendsen, 2017[64] | 0 | 0 | 2 | 0 | 2 | High risk |
| Lerena, 2016[65] | 1 | 0 | 2 | 0 | 3 | High risk |
| PWID |  |  |  |  |  |  |
| Aisyah, 2018[66] | NR | NR | NR | 1 | 1 | Low risk |
| Hope, 2016[67] | NR | NR | NR | 2 | 2 | Low risk |
| Hope, 2015[30] | NR | NR | NR | 2 | 2 | Low risk |
| Valencia, 2018[68] | NR | NR | NR | 0 | 0 | High risk |
| Folch, 2016[32] | NR | NR | NR | 1 | 1 | Low risk |
| Leon, 2016[69] | NR | NR | NR | 2 | 2 | Low risk |
| Weill-Barillet, 2016[28] | NR | NR | NR | 2 | 2 | Low risk |
| Sypasa, 2017[70] | NR | NR | NR | 0 | 0 | High risk |
| Sheka, 2014[29] | NR | NR | NR | 0 | 0 | High risk |
| Derks, 2018[71] | NR | NR | NR | 2 | 2 | Low risk |
| Tarjan, 2017[31] | NR | NR | NR | 2 | 2 | Low risk |
| Handanagic, 2016[72] | NR | NR | NR | 2 | 2 | Low risk |
| Kaberg, 2017[73] | NR | NR | NR | 0 | 0 | High risk |
| Keegan, 2017[74] | NR | NR | NR | 0 | 0 | High risk |
| Skocibusic, 2016[75] | NR | NR | NR | 0 | 0 | High risk |
| Svendsen, 2017[76] | NR | NR | NR | 0 | 0 | High risk |
| Nosotti, 2016[77] | NR | NR | NR | 0 | 0 | High risk |
